# Calcified lymph nodes and systemic sclerosis

**DOI:** 10.31138/mjr.29.2.97

**Published:** 2018-06-29

**Authors:** Fotini Angelopoulou, Pantelis Kraniotis, Stamatis-Nick Liossis, Dimitrios Daoussis

**Affiliations:** 1 Undergraduate Student, University of Patras Medical School,; 2 Departments of Radiology and; 3 Rheumatology, Patras University Hospital, University of Patras Medical School, Patras, Greece

**Keywords:** systemic sclerosis, scleroderma, lymphadenopathy

## CLINICAL IMAGE

**Figure 1.**
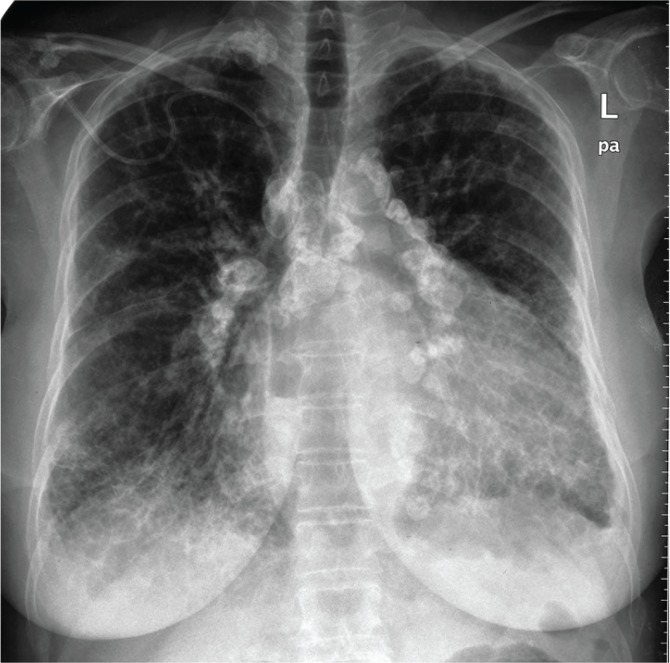


**Figure 1** depicts a chest X-ray of a 55-year-old female with a long history of diffuse systemic sclerosis. The patient first presented to the Rheumatology Clinic in 1984 with Raynaud’s, sclerodactyly, cutaneous telangiectasias and recurrent ischemic digital ulcers with positive anti-Scl70 antibodies. Based on the clinical and laboratory findings a diagnosis of diffuse systemic sclerosis (SSc) was made.

Interestingly, in addition to all the above mentioned typical features of SSc, the patient also had a palpable, hard, supraclavicular lymph node from the beginning of her illness. An extensive work up was performed to rule out malignancy. Chest CT revealed extensive mediastinal lymphadenopathy. Notably, lymph nodes were calcified in an “eggshell” pattern. Supraclavicular lymph node biopsy ruled out malignancy; moreover, no evidence of granulomatous disease was found.

Throughout her 30 years of follow-up, the patient slowly but steadily deteriorated. She was treated with D-penicillamine, methotrexate and rituximab respectively; none of which had any effect on her radiographic findings. She never had SSc-related calcifications. She developed interstitial lung disease, gastrointestinal involvement with esophageal dysmotility and severe recurrent digital ulcers that lead to autoamputation of most of her fingers.

At the later stages of the disease, she also developed heart failure and pulmonary arterial hypertension. Calcified mediastinal lymph nodes were always visible on plain x-rays during the course of her illness without any evidence of change over time.

Her chest x-ray presented herein, has many findings:

- A reticular pattern is noted in both lungs, more prominent in the lower lung zones. The appearance is consistent with interstitial lung disease in the context of SSc;
- The esophagus is dilated, with the presence of an air-fluid level, a common finding in SSc;
- Abnormal cardiothoracic ratio, with a markedly enlarged heart due to heart failure;
- There is extensive mediastinal and hilar lymphadenopathy; the lymph nodes are calcified in an “eggshell” pattern. There are also similarly calcified supraclavicular lymph nodes on the right.

The exact cause of calcific lymphadenopathy in this particular case was never proven. The differential diagnosis of calcific mediastinal lymphadenopathy is broad and includes tuberculosis, sarcoidosis, lymphoma, metastasis from thyroid cancer as well as osteogenic sarcoma, amyloidosis and Castleman disease. The extremely long history virtually rules out malignant or infectious causes. Sarcoidosis is included in the differential diagnosis, but the biopsy was not supportive; the patient also never had any typical features of this disease. Occupational exposure such as silicosis is one of the causes of “eggshell” calcific mediastinal lymphadenopathy. The patient was a dental technician, a profession known to associate with exposure to silica. Interestingly, silica is also a well known environmental trigger for SSc. We believe that the most likely scenario for our patient is that occupational exposure to silica triggered both SSc and lymphadenopathy. Of note, there is similar published case report in the literature.^1^
